# Different Types of Connections Between the Thalamus and Vestibular Nucleus in the Human Brain

**DOI:** 10.3390/jcm14217551

**Published:** 2025-10-24

**Authors:** Sang-Soo Lee, Seo-Yoon Park, Sang-Seok Yeo

**Affiliations:** 1 Department of Health, Graduate School, Dankook University, Cheonan 31116, Chungnam, Republic of Korea; lsspt2531@naver.com; 2 Department of Physical Therapy, College of Health and Welfare, Woosuk University, Wonju 55338, Jeonbuk, Republic of Korea; pgy0614@hanmail.net; 3 Department of Physical Therapy, College of Health Sciences, Dankook University, Cheonan 31116, Chungnam, Republic of Korea

**Keywords:** vestibulothalamic tract, diffusion tensor imaging, vestibular nuclei, thalamic nuclei, neuroanatomical connectivity

## Abstract

**Background/Objectives:** The vestibulothalamic tract (VTT) serves as a crucial pathway transmitting vestibular information from the brainstem nuclei to the thalamus, where integration with other sensory modalities occurs. This study aimed to investigate the structural connectivity between three vestibular nuclei and three thalamic nuclei in the human brain using diffusion tensor imaging (DTI) tractography. **Methods:** Twelve healthy adults underwent DTI to visualize vestibulothalamic connections using probabilistic tractography. **Results:** Results revealed distinct patterns of connectivity: the lateral vestibular nucleus (LVN) exhibited the highest reconstruction rates to both the ventral posterolateral (95.8%) and ventral posteromedial (83.3%), while the medial vestibular nucleus (MVN) showed the strongest connection to the ventral intermediate (75.0%). All vestibulothalamic tracts predominantly passed through the tegmentum of the midbrain, with limited or absent contributions from the tectum. **Conclusions:** These findings indicate differential roles of vestibular nuclei in relaying information to thalamic targets, with the LVN showing preferential projections to sensory relay nuclei and the MVN contributing to motor-related thalamic regions. Such insights may have important implications for the diagnosis and treatment of vestibular disorders, as well as for advancing anatomical research. These findings provide anatomical insights that may help explain symptoms of vestibular and thalamic lesions and guide rehabilitation strategies for balance and gaze control disorders.

## 1. Introduction

The vestibular nuclei comprise a group of nuclei located in the brainstem that play a crucial role in processing information related to balance, spatial orientation, and coordination of eye movements [1,2]. This complex receives input from the vestibular system, a sensory system in the inner ear responsible for detecting changes in head position and movement [2]. There are four types of vestibular nuclei (the superior, inferior, medial, and lateral), all of which play a role in controlling head, neck, and eye movements [3]. The vestibular nuclei in the brainstem are primarily connected to the cerebellum and various thalamic nuclei through the vestibulothalamic tract (VTT) [4]. The primary function of the VTT is to transmit information from the vestibular nuclei to the thalamus. This information is crucial for integrating the vestibular signals with other sensory information, including visual and proprioceptive inputs [4,5].

The thalamus plays a crucial role in relaying sensory and motor signals to the cerebral cortex, further contributing to various functions in the central nervous system. It acts as a relay station for sensory information from various sensory organs to corresponding areas of the cerebral cortex [6]. In addition to relaying sensory information, the thalamus is also involved in motor control, receiving input from the cerebellum, vestibular nucleus, and basal ganglia, sending motor signals to the motor cortex, and aiding in the coordination of voluntary movements [7]. Thalamocortical connections are bidirectional, meaning that information flows from the thalamus to the cortex, and vice versa. These connections are vital for integrating and coordinating sensory inputs, thus allowing the brain to form coherent perceptions of the surrounding environment [6,8,9].

The term “thalamic nucleus” refers to a group of cell bodies (neurons) located within the thalamus, which is a core structure of the brain. It comprises several individual nuclei, each with a specific function, which are broadly categorized into relay nuclei, which transmit sensory information to the cortex, and association nuclei, which are involved in information integration and processing [6,10]. In addition to sensory processing, the thalamus plays a role in relaying functions such as movement, consciousness, memory, and emotion [7,11]. Nuclei related to the vestibular system include the ventral posterolateral (VPL), ventral posteromedial (VPM), and ventral intermediate (VI). Multiple projections between the vestibular and thalamic nuclei have also been reported [4,12,13].

In this context, the present study aimed to use diffusion tensor imaging (DTI) analysis to examine the various connections between the vestibular and thalamic nuclei in the human brain, and to further reveal their relay pathways. Clinically, damage to vestibulothalamic connections has been linked to imbalance and spatial disorientation observed in thalamic or brainstem lesions, providing anatomical rationale for balance rehabilitation [6,7].

## 2. Materials and Methods

### 2.1. Subjects

Twelve healthy subjects (males: 6, females: 6, mean age: 35.8 years, range: 26–52 years) with no history of neurological or musculoskeletal disease were enrolled in this study following recruitment from D University. The inclusion criteria were: (1) no diagnosis associated with vestibular function, (2) no history of cerebellar injury, and (3) no history of neurological, cognitive, or musculoskeletal dysfunction. The exclusion criteria were as follows: (1) prior diagnosis of musculoskeletal or neurological problems or (2) past history of brain injury. All participants provided informed consent prior to DTI tractography. This study was approved by the institutional review board of D-University prior to initiation. (2024-03-024-003).

### 2.2. Diffusion Tensor Image Tractography

DTI data were acquired using a 6-channel head coil on a 3T Philips Gyroscan Intera (Philips, Best, The Netherlands) with single-shot echo-planar imaging. For each of the 32 non-collinear diffusion sensitizing gradients, 67 contiguous slices were acquired parallel to the anterior commissure–posterior commissure line. The imaging parameters were as follows: acquisition matrix = 96 × 96, reconstructed matrix = 192 × 192 matrix, field of view = 240 × 240 mm^2^, TR = 10,398 ms, TE = 72 ms, parallel imaging reduction factor (SENSE factor) = 2, EPI factor = 49 and b = 1000 s/mm^2^, NEX = 1 and a slice thickness of 2.5 mm with no gap (acquired voxel size: 1.3 × 1.3 × 2.5 mm^3^) [14].

### 2.3. Fiber Tracking

Diffusion-weighted imaging data were analyzed using the Oxford Centre for Functional Magnetic Resonance Imaging of the Brain (FMRIB) Software Library (FSL v5.0; www.fmrib.ox.ac.uk/fsl, accessed on 5 March 2024). Affine multiscale two-dimensional registration was used to correct head motion and eddy current-induced image distortion. Fiber tracking was performed using a probabilistic tractography method based on a multiple tensor model, with tractography routines implemented in FMRIB Diffusion (5000 streamline samples, 0.5 mm step lengths, curvature thresholds = 0.2).

**Figure 1:**
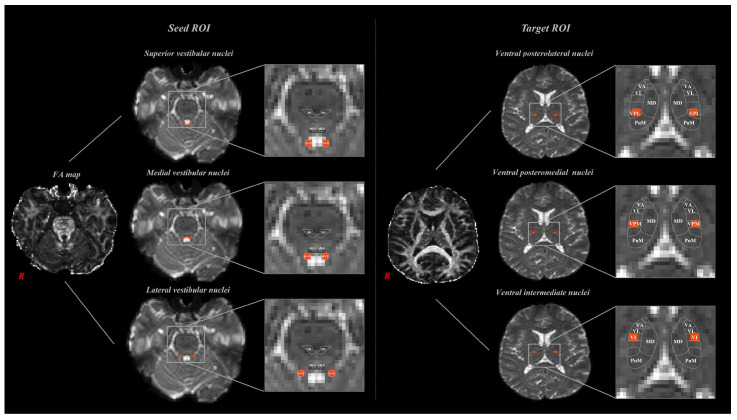
Seed region of interest (ROI) and Target ROIs set in magnetic resonance imaging (MRI) analysis. Seed ROIs were placed in the vestibular nuclei (SuVN, MVN, LVN) in the brainstem, and target ROIs were placed in the thalamic nuclei (VPL, VPM, VI).

VTTs were determined by selecting fibers with nine tracts through three seed regions of interest (ROI), and three target ROIs. The seed ROIs of the superior vestibular nuclei (SVN), medial vestibular nuclei (MVN), and lateral vestibular nuclei (LVN) were located in the medulla and pons of the brainstem [3]. The seed ROIs included the superior vestibular nuclei (SVN), medial vestibular nuclei (MVN), and lateral vestibular nuclei (LVN) of the medulla and pons of the brainstem [3]. The target ROIs, which included the ventrobasal complex, ventral posterolateral nucleus (VPL), ventral posteromedial nucleus (VPM), and ventral intermediate nucleus (VI), are located caudally in the thalamus (Figure 1) [15]. A total of 5000 samples were generated from a seed voxel, and the results were visualized at a minimum of one streamline for each voxel. Based on previous anatomical and neuroimaging studies [3,15], each region of interest (ROI) was placed at the most reliable and reproducible anatomical locations for the vestibular and thalamic nuclei. These prior works have consistently defined the spatial boundaries and relative coordinates of the superior, medial, and lateral vestibular nuclei as well as the VPL, VPM, and VI thalamic nuclei, providing robust anatomical validation for our ROI positioning. Tractography was performed separately for each hemisphere, and reconstruction rates were averaged across sides to provide representative values.

### 2.4. Statistical Analysis

Descriptive statistical analysis was applied to determine differences in neural pathways and connectivity.

## 3. Results

**Figure 2:**
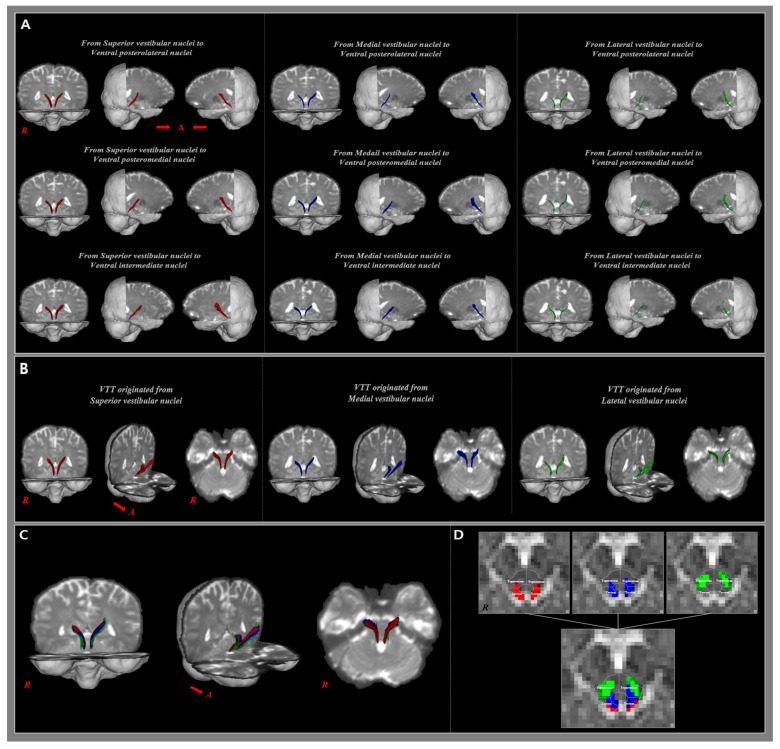
(**A**) The 9 analyzed vestibulothalamic tracts (VTT) are shown in the coronal plane and both sides of the sagittal plane. Color coding: Red—SuVN projections, Blue—MVN projections, Green—LVN projections. (**B**) Each of the 3 VTTs originating from the same seed ROI are shown combined. (**C**) A total of 9 VTTs are shown to be combined in the coronal plane and both sides of the sagittal plane. (**D**) The areas passing through the tectum and tegmentum at the mid-brain level are represented with the VTTs originating from the same seed ROI and 9 total VTTs, respectively.

In the current study, we implemented nine tracts by selecting three seed ROIs (the SuVN, MVN, and LVN) and three target ROIs (the VPL, VPM, and VI). Each of the nine VTT pathways originates from the pontine VN and terminates on the caudal side of the thalamic nucleus (Figure 2). The reconstruction rate of the VTT to the VPL was the highest in the LVN (95.8%), followed by the SuVN (62.5%) and MVN (41.7%). The reconstruction rate of the VTT to the VPM was also highest in the LVN (83.3%), followed by the SuVN (75.0%) and MVN (58.3%). The reconstruction rate of the VTT to the VI was the highest in the MVN (75.0%), followed by the SuVN (70.8%) and MVN (66.7%) (Table 1).

The analyzed VTT was connected to the thalamic nucleus through the tegmentum and tectum of the midbrain, and the rate of passage through the tegmentum was higher than through the tectum. The VTT originating from the SuVN, MVN, and LVN passes through the tegmentum area of the midbrain, and is connected to the VPL, VPM, and VI areas. In contrast, for VTTs from the SuVN, the proportion connected to the thalamus via the tectum was 33.3% for the VPL, 38.9% for the VPM, and 41.2% for the VI. For the VTTs from the MVN, the proportion connected to the thalamus via the tectum was 20.0% for the VPL, 7.1% for the VPM, and 22.2% for the VI. However, among VTTs originating from the LVN, there were no connections to the thalamus via the tectum (Table 2).

## 4. Discussion

The purpose of this study was to analyze the VTT between the three vestibular nuclei and three thalamic nuclei using DTI, and to compare the characteristics of each connectivity. The results of this study showed that all three vestibular nuclei had a certain level of connectivity with the thalamus. The vestibular nuclei are located in the brainstem, specifically within the pons and medulla oblongata, and there are four main nuclei: the superior, inferior, medial, and lateral VN [3]. This study examined the connections between the VPL, VPM, and VI thalamic nuclei and three vestibular nuclei: the lateral, medial, and superior nuclei. The LVN primarily aids the vestibulospinal reflexes to maintain posture and balance, with mediation by the paravertebral and proximal extensor muscles [16,17]. The MVN facilitates the vestibulo-ocular reflex and ensures clear vision during horizontal head movement [18]. Meanwhile, the SVN is associated with the perception of gravity and body movement [3]. Given this knowledge, the present study aimed to understand how these thalamic nuclei connect with the vestibular nuclei, in order to shed light on the neural pathways involved in sensory processing and motor coordination related to balance, posture, and vision.

The VPL area is known to be a major connection area n vestibulothalamic projections; however, it is not specialized only for vestibular function, showing connectivity with other peripheral sensory and cortical areas [19]. The strong LVN–VPL/VPM connectivity observed may reflect vestibulo-somatosensory integration critical for postural control and spatial perception [19,20]. Conversely, the MVN–VI connection likely contributes to thalamic circuits regulating coordinated movement and tremor modulation [21,22]. Overall, the results of this study showed that the was LVN was then vestibular nucleus with the highest connectivity with the VPL, while the SuVN and MVN showed relatively low connectivity (Table 2). The neurological connectivity between the vestibular nuclei and VPM area remains poorly understood. However, several studies have shown that VPM neurons respond directly to vestibular nerve stimulation, as well as to rotational and translational movements associated with vestibular sensation [4,20]. In this study, the VPM thalamic nucleus also showed the highest connectivity with the LVN, followed by the SuVN and MVN. The VI plays a central role in motor control in the thalamic nucleus, and is particularly involved in the coordination of muscle activity and limb movement during voluntary movements of the human body [20,21]. The VI is also considered a target area for deep brain stimulation to alleviate tremor symptoms in patients with Parkinson’s disease [22]. Conversely, relatively little is known about the neurological connectivity with the vestibular region. In the present study, the MVN showed the highest connectivity with the VI, followed by the SuVN and LVN.

In this study, we found that all connection paths between the three vestibular nuclei and the thalamic nucleus passed through the tegmental area of the midbrain, whereas connectivity with the tectum was relatively low or absent in some cases. The tegmentum is the ventral part of the midbrain, and is primarily involved in visual and auditory information processing, motor control, and autonomic nervous system function [23]. In 2008, Zwergal et al. [24] investigated the correlation between ocular tilt reaction problems and interstitial nuclei of Cajal in patients with brainstem lesions. Our results confirmed that 96% of the patients with ocular tilt reactions had damage to the paramedian posterior tegmentum related to the medial longitudinal fascicle. The tectum refers to the dorsal midbrain, which plays an important role in processing visual and auditory information. However, little research has been conducted on the anatomical and functional relationships between the tectum and vestibular system. In the present study, most vestibulothalamic fibers traversed the midbrain tegmentum rather than the tectum. The tegmentum is known to function as a major relay site integrating vestibular information with oculomotor and postural control systems via the periaqueductal and pontine regions. In contrast, the tectum primarily mediates visual–vestibular feedback for gaze stabilization and head–eye coordination [23,25,26]. Therefore, the dominance of tegmental pathways observed in this study supports their critical role in combining vestibular, ocular, and postural information for spatial orientation and motor control. Nevertheless, several studies have shown that the tectum, along with the visual cortex, is important for gaze stabilization related to vestibular function [25,26]. Therefore, the tegmentum area of the midbrain is thought to function as the main relay pathway in the connectivity between the vestibular nuclei and the thalamic nuclei, while the tectum is believed to be able to contribute to some extent.

Several previous studies using animal models or neuroimaging analysis methods in the human brain have reported on the neurological connectivity between the vestibular and thalamic nuclei. In 1999, Shiroyama et al. [27] reported on the connectivity between each vestibular and thalamic nucleus in rats using the anterograde axonal tracer method. Injections into the SuVN and lateral VN showed connectivity with various thalamic nuclei, including the VPL, VPM, and VL. Injections into the MVN showed major connectivity with the VPM, and minor connectivity with the VPL and posterior thalamic nucleus. In 2002, Bácskai et al. [28] reported on the ascending and descending projection pathways of the lateral VN in rats. The ascending projection of the lateral VN passes through the medial longitudinal fasciculus to the thalamus, and predominantly terminates at the caudal one-third of the thalamus. The VPM is the area with the highest density. Additionally, previous studies have suggested the existence of an ipsilateral VTT in the human brain [29,30,31,32]. Based on the results of previous studies, it can be assumed that the ipsilateral VTT originates around the medial lemniscus and is connected to the posterolateral thalamus [29]. In 2018, Jang and Kwon [30] investigated and reported the anatomical characteristics of the ipsilateral VTT in the human brain using DTI. The reconstructed ipsilateral VTT was connected posterolaterally to the upper pons and anteromedially to the upper midbrain, situated between the vestibular and lateral thalamic nuclei.

In conclusion, this study analyzed and compared the VTT from three vestibular nuclei (the SVN, MVN, and LVN) to the VPL, VPM, and VI thalamic nuclei in young adults. The results showed that the VTT from the three vestibular nuclei to the thalamus were different, but all were connected through the tegmentum and tectum regions of the midbrain. Therefore, this study provides basic data on the processes by which peripheral vestibular information is transmitted to the central nervous system, and can serve as neurophysiological data for the central vestibular system. Understanding these connectivity patterns provides a neuroanatomical framework for interpreting balance deficits, dizziness, and gaze instability observed in patients with thalamic or vestibular lesions. This knowledge can inform vestibular rehabilitation strategies emphasizing postural alignment, sensory reweighting, and gaze stabilization exercises. In addition, mapping the VI nucleus connections offers potential guidance for neurosurgical procedures, including deep brain stimulation (DBS), aimed at modulating tremor and vestibular-related motor dysfunctions. However, this study has several limitations that should be considered. First, it targeted only a small number of adults; therefore, the results cannot be generalized. This limitation primarily stems from methodological constraints: the small anatomical size of the vestibular nuclei and the high-resolution requirements of probabilistic tractography made it difficult to recruit a large number of participants. Future studies using larger cohorts or multi-center datasets will be valuable for validation. Secondly, it was difficult to locate an accurate ROI because of the diminutive size of the VN and thalamic nucleus. Finally, only the connectivity between the ipsilateral VN and the thalamic nucleus was analyzed, while the contralateral connection was not analyzed. Therefore, in future studies, it will be necessary to study the bilateral connectivity between the VN and thalamic nucleus by recruiting a larger number of subjects of various ages.

## Figures and Tables

**Table 1:** Comparison of reconstruction rates between the vestibular and thalamic nuclei.

ROIs	VPL		VPM		VI	
	Recon	Non-Recon	Recon	Non-Recon	Recon	Non-Recon
SuVN	15	9	18	6	17	7
	(62.5%)	(37.5%)	(75.0%)	(25.0%)	(70.8%)	(29.2%)
MVN	10	14	14	10	18	6
	(41.7%)	(58.3%)	(58.3%)	(41.7%)	(75.0%)	(25.0%)
LVN	23	1	20	4	16	8
	(95.8%)	(4.2%)	(83.3%)	(16.7%)	(66.7%)	(33.3%)

ROIs: region of interest; SuVN: superior vestibular nuclei; MVN: medial vestibular nuclei; LVN: lateral vestibular nuclei; VPL: ventral posterolateral; VPM: ventral posteromedial; VI: ventral intermediate; Recon: reconstructed; Non-Recon: Non-reconstructed.

**Table 2:** Tectum and tegmentum passage rates of the 9 VTT.

ROIs	VPL		VPM		VI	
	Tegmentum	Tectum	Tegmentum	Tectum	Tegmentum	Tectum
SuVN	15/15	5/15	18/18	7/18	17/17	7/17
	(100.0%)	(33.3%)	(100.0%)	(38.9%)	(100.0%)	(41.2%)
MVN	10/10	2/10	14/14	1/14	18/18	4/18
	(100.0%)	(20.0%)	(100.0%)	(7.1%)	(100.0%)	(22.2%)
LVN	23/23	0/23	20/20	0/20	16/16	0/16
	(100.0%)	(0.0%)	(100.0%)	(0.0%)	(100.0%)	(0.0%)

ROIs: regions of interest; SuVN: superior vestibular nuclei; MVN: medial vestibular nuclei; LVN: lateral vestibular nuclei; VPL: ventral posterolateral; VPM: ventral posteromedial; VTT: vestibulothalamic tract; VI: ventral intermediate.

## Data Availability

The data presented in this study are available on request from the corresponding author due to privacy.

## Abbreviations

The following abbreviations are used in this manuscript:

VTT	vestibulothalamic tract
DTI	diffusion tensor imaging
VPL	ventral posterolateral
VPM	ventral posteromedial
VI	ventral intermediate
FMRIB	Functional Magnetic Resonance Imaging of the Brain
SVN	superior vestibular nuclei
MVN	medial vestibular nuclei
LVN	lateral vestibular nuclei
